# Expression of Prostate-Specific Membrane Antigen in Lung Cancer Cells and Tumor Neovasculature Endothelial Cells and Its Clinical Significance

**DOI:** 10.1371/journal.pone.0125924

**Published:** 2015-05-15

**Authors:** Hai-long Wang, Shao-shan Wang, Wen-hui Song, Yi Pan, Hai-peng Yu, Tong-guo Si, Yong Liu, Xiao-nan Cui, Zhi Guo

**Affiliations:** 1 Department of Interventional Treatment, Tianjin Medical University Cancer Institute and Hospital, National Clinical Research Center for Cancer Laboratory of Cancer Prevention and Therapy, Tianjin, China; 2 Department of Oncology, Dagang Hospital of Binhai New Area, Tianjin, China; 3 Department of Urology, Tianjin First Center Hospital, Tianjin, China; 4 Department of Pathology, Tianjin Medical University Cancer Institute and Hospital, Tianjin, China; 5 Department of Internal Medicine, University of Arkansas Medical Sciences, Little Rock, Arkansas, United States of America; 6 Department of Oncology, the First Affiliate Hospital of Dalian Medical University, Dalian, China; University of Sassari, ITALY

## Abstract

**Background:**

Prostate-specific membrane antigen (PSMA) has been found in tumor neovasculature endothelial cells (NECs) of non-prostate cancers and may become the most promising target for anti-tumor therapy. To study the value of PSMA as a potential new target for lung cancer treatment, PSMA expression in non-small cell lung cancer (NSCLC) and small cell lung cancer (SCLC) tissues and its relationship with clinicopathology were investigated in the current study.

**Methods:**

Immunohistochemistry was used to detect PSMA expression in a total of 150 lung specimens of patients with lung cancer. The data were analyzed using univariate and multivariate statistical analyses.

**Results:**

The percentages of NSCLC patients who had PSMA (+) tumor cells and PSMA (+) NECs were 54.02% and 85.06%, respectively. The percentage of patients younger than 60 years old who had PSMA (+) tumor cells was 69.05%, which was significantly greater than the percentage of patients aged 60 years or older (40.00%, p<0.05). A significant difference was observed in the percentage of NSCLC patients with PMSA (+) NECs and stage I or II cancer (92.98%) and those patients with stage III or IV cancer (76.77%). In the SCLC tissues, NEC PSMA expression (70.00%) did not differ significantly from NSCLC. SCLC tumor cells and normal lung tissues cells were all negative. There was no significant correlation between the presence of PSMA (+) NECs in SCLC patients and the observed clinicopathological parameters.

**Conclusions:**

PSMA is expressed not only in NECs of NSCLC and SCLC but also in tumor cells of most NSCLC patients. The presence of PSMA (+) tumor cells and PSMA (+) NECs in NSCLC was negatively correlated with age and the clinicopathological stage of the patients, respectively.

## BACKGROUND

The most recent statistics show that lung cancer has become the leading cause of all cancer-related deaths [1]. Targeted therapy, particularly targeted anti-vascular therapy, may become an effective new treatment for lung cancer. In targeted therapy, the ideal targets should exist specifically in tumor cells and tumor neovasculature endothelial cells (NECs). Prostate-specific membrane antigen (PSMA) is a type II transmembrane protein, and its encoding gene is located on 11p11-p12 [2–7]. PSMA contains three regions: an intracellular domain of 19 amino acids, a transmembrane domain of 24 amino acids, and an extracellular domain of 707 amino acids [8,9]. This structure allows signals from cell surface proteins to be transduced into cells [10,11]. Unlike other prostate-related antigens, PSMA is not secreted into the circulatory system [12,13]. Instead, PSMA regulates tumor cell invasion and tumor angiogenesis by modulating integrin signal transduction in endothelial cells [14]. PSMA is not only widely expressed in prostate cancer cells but is also present in NECs of non-prostate tumors such as non-small cell lung cancer (NSCLC) [15–18].

Unlike other tumor neovasculature targets, PSMA is expressed specifically in tumor NECs and is not expressed in the endothelial cells of normal tissues. Common vascular targets, such as vascular endothelial growth factor (VEGF), endoglin and integrins, are expressed in the vascular cells of both normal tissue and tumor tissue. Hence, PSMA may become the most promising target for anti-tumor therapy due to its distinct structure and functions.

To study the value of PSMA as a potential new target for lung cancer treatment, we measured its expression in lung cancer tissues and then correlated its expression with patient age at the time of diagnosis, gender, clinical stage, pathological type, primary tumor size, and lymph node metastasis. To our knowledge, no related studies have been previously reported in the literature.

## MATERIALS AND METHODS

### 1. Tissue samples

This study was approved by the Ethics Committee of the First Affiliate Hospital of Dalian Medical University (KY2014-08, S1 Certificate). Paraffin-embedded lung cancer tissue and normal lung tissue samples were obtained from the Pathology Department of the Tianjin Medical University Cancer Institute & Hospital, and the ethics committee of the Tianjin Medical University Cancer Institute & Hospital waived the need for the written informed consent of these Paraffin-embedded samples (2012BWZ006, S2 Certificate). The samples obtained were anonymized and de-identified prior to analysis. The samples consisted of surgically resected tissues from hospitalized patients during the period between January 2007 and December 2012. Detailed clinical data and follow-up information were available for all enrolled lung cancer patients. This study collected a total of 117 lung cancer tissue specimens, including 87 cases of NSCLC (30 cases of squamous cell carcinoma, 29 cases of adenocarcinoma, and 28 cases of large cell carcinoma) and 30 cases of small cell lung cancer (SCLC), and 33 normal lung tissue specimens.

### 2. Immunohistochemistry

Conventional immunohistochemical methods were used. Paraffin-embedded tissue specimens were sectioned into 4 mm-thick slices, heated at 65°C for 2 hours, and deparaffinized in xylene for 2 hours. After deparaffinization and gradient ethanol hydration, the sections were pre-treated with citric acid antigen retrieval solution (0.01 M citric acid, *pH 6*.*0*) at 120°C. Hydrogen peroxide was then added dropwise to the slides, which were incubated for 10 minutes at room temperature. After washing, the primary antibody was added dropwise to the slides and incubated at 4°C overnight. The primary antibodies used were: PSMA monoclonal antibody (Abcam Inc., Cambridge, UK) and CD31 monoclonal antibody (Proteintech Group, Chicago, IL, USA). After washing, the second antibody, goat anti-mouse HRP-IgG (Zhongshan Goden Bridge Biotechnology CO., Ltd, Beijing, China), was added dropwise and incubated for 40 minutes at room temperature. After washing, freshly prepared 3,3'-diaminobenzidine (DAB) chromogenic reagent (Zhongshan Goden Bridge Biotechnology CO., Ltd, Beijing, China) was added dropwise to the slides. After 3–5 minutes of chromogenic reaction, the reaction was terminated, followed by hematoxylin staining, alcohol dehydration, xylene clarification, and neutral resin mounting.

To determine the expression of PSMA in tumor NEC, we used CD31 expression as a reference. Prostate cancer tissues positive for PSMA were used as positive controls, and PBS buffer was used to substitute for the primary antibody solution for negative controls. For PSMA-positive lung cancer tissue samples, we performed a re-examination using a PSMA monoclonal antibody produced by an independent company (Proteintech Group, Chicago, USA) to verify the reliability of the experimental results.

Slides were analyzed by light microscopy (OLYMPUS MODEL BX53F, Tokyo, Japan). We observed 3 fields per section at 400× magnification, and positive cell numbers were counted in 100 random tumor cells in every field. The staining intensity and the mean percentage of positive cells were used to determine the expression of the proteins in a section. All of these counts were blindly performed in at least 3 randomly chosen sections. The staining intensity and percentage of positive cells were scored semi-quantitatively by two pathologists. The intensity of staining was classified into four categories [19]: "0" for no brown particle staining (none of the cells displayed immunostaining), "1" for light brown particles, "2" for moderate brown particles, and "3" for dark brown particles. The percentage of positive cells was divided into four groups [20]: "0" for <5% positive cells, "1" for 6%-30% positive cells, "2" for 31%-60% positive cells, and "3" for ≥60% positive cells. The sum of the intensity and percentage of positive staining was used to determine positive (score ≥ 1) or negative (score <1) expression of PSMA.

### 3. Statistical analysis

The relationships between PSMA and patient age at diagnosis, pathological type, and clinical stages were analyzed using a chi-square test. Experimental data were statistically analyzed using the SPSS17.0 statistical software package (SPSS 17.0, Chicago, IL, USA). *P<0*.*05* was considered statistically significant. A portion of the data was analyzed using the SAS 9.3 statistical analysis system (SAS Institute Inc. Cary, NC, USA).

## RESULTS

### 1. PSMA expression in NSCLC tissue

**Fig 1:**
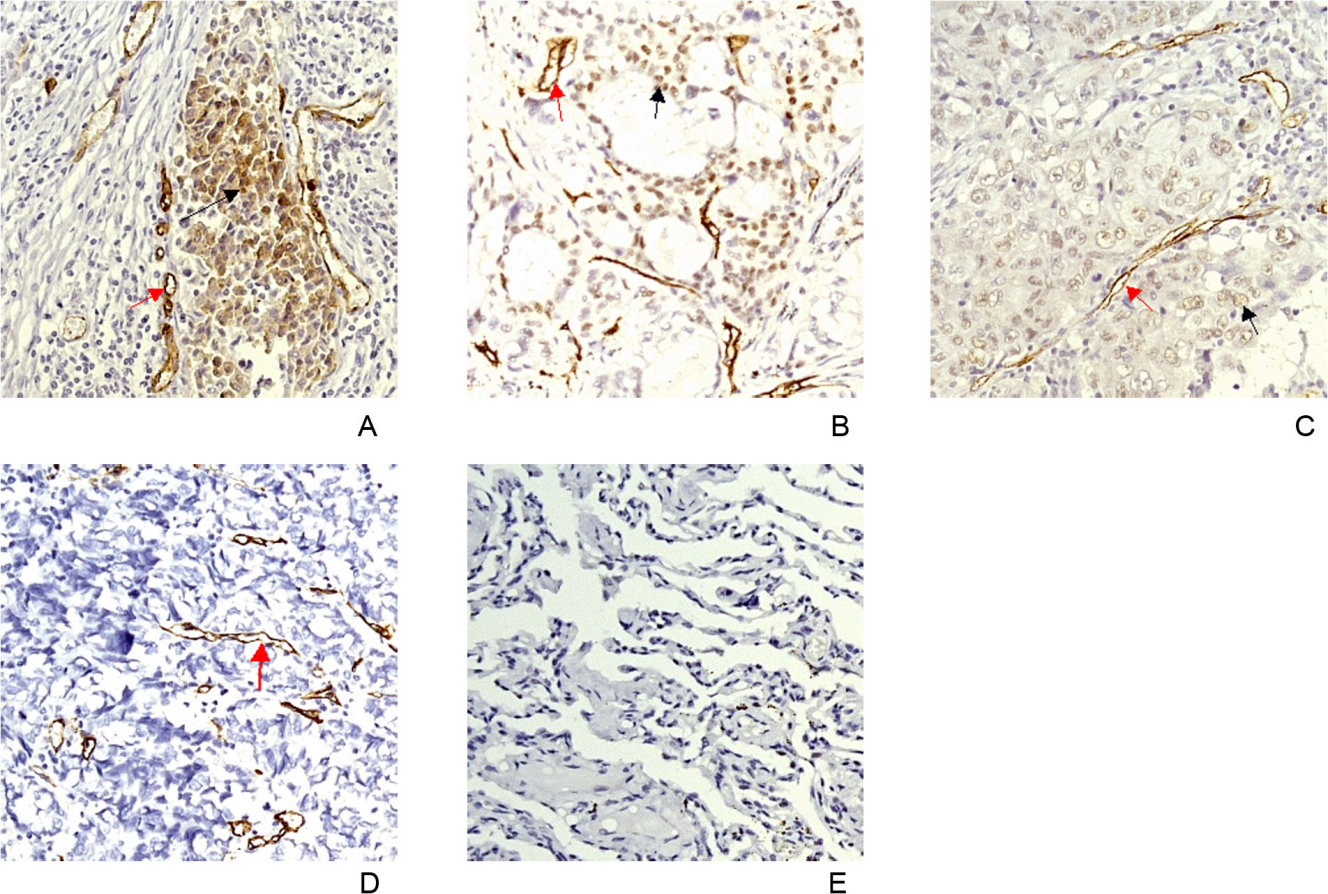
PSMA expression in NSCLC tissues (400×). Red arrows indicate PSMA-positive neovasculature endothelial cells (NEC) of tumor tissues. Black arrows indicate PSMA-positive tumor cells. A. lung squamous cell carcinoma. B. lung adenocarcinoma. C. large cell lung carcinoma. D. small cell lung carcinoma. E. normal lung tissues.

PSMA expression was detected using immunohistochemistry in NSCLC tissue specimens, which were collected from 87 NSCLC cases, including 30 cases of squamous cell carcinoma, 29 cases of adenocarcinoma, and 28 cases of large cell carcinoma. PSMA expression was confirmed in NECs of NLCSC. Furthermore, we discovered for the first time that PSMA was expressed in the tumor cells of more than half of the NSCLC patients (Fig 1A, 1B and 1C and Table 1). Of the 87 NSCLC cases, 74 had PSMA expression in tumor NECs, with a positive rate of 85.06% (74/87); 47 displayed PSMA expression in the tumor cells, with a positive rate of 54.02% (47/87); 40 cases had both PSMA-positive tumor cells and tumor NECs (45.98%, 40/87).

**Table 1:** PSMA expression in non-small cell lung cancer.

Factor	n	Tumor cell p value positive(%)	Neovasculature p value positive(%)
**Age**			
<60	42	29(69.05%)*p = 0*.*007*	33(78.57%)*p = 0*.*101*
≥60	45	18(40.00%)	41(91.11%)
**Gender**			
Male	56	28(50.00%)*p = 0*.*312*	42(75.00%)*p = 0*.*182*
Female	31	19(61.29%)	27(87.10%)
**TNM stage**			
I+II	57	33(57.89%)*p = 0*.*318*	53(92.98%)*p = 0*.*03*
III+IV	30	14(46.67%)	23(76.77%)
**Tumor size**			
≤3cm	24	12(50.00%)*p = 0*.*642*	20(83.33%)*p = 0*.*781*
>3cm	63	35(55.56%)	54(85.71%)
**Lymph node**			
positive	39	21(53.85%)*p = 0*.*976*	31(79.49%)*p = 0*.*189*
negative	48	26(54.17%)	43(89.58%)
**Pathological type**			
Squamous	30	15(50.00%)*p = 0*.*303*	28(93.33%)*p = 0*.*169*
Adeno	29	19(65.52%)	22(75.86%)
large cell	28	13(46.43%)	24(85.71%)

The data shows a clear positive correlation between clinical stage and the presence of PSMA-positive NECs of NSCLC. In 57 stage I and II patients, the percentage with PSMA-positive NECs was 92.98%; in 30 stage III and IV patients, the percentage was 76.77%. SAS statistical software was used for statistical trend analysis, and the results suggested that the percentage of stage I and II patients with PMSA-positive NECs was significantly higher than that of stage III and IV patients (*p = 0*.*03*). However, in contrast, there was no significant correlation between the percentage of patients with PSMA-expressing tumor cells and clinical stage (Table 1, *p = 0*.*318*).

As shown in the table, the percentage of patients with PSMA-positive NSCLC tumor cells correlated with patient age at diagnosis. The percentage of patients younger than 60 years with PMSA-positive tumor cells was significantly higher than that of patients aged 60 years or older (69.05% versus 40.00%, *p = 0*.*007*). However, there was no correlation between age and the percentage of patients with PMSA-positive NECs in NSCLC (*p = 0*.*101*).

In addition, immunohistochemistry results suggested that there were no statistically significant correlations between the percentage of patients with PSMA-positive NECs or/and tumor cells and gender, pathological type of NSCLC, size of primary tumor, or presence or absence of lymph node metastasis (Table 1).

### 2. PSMA expression in SCLC tissues

30 SCLC lung tissue samples were involved in this study. PSMA expression was detected only in the NECs (70.00% of patients) and not in tumor cells (Fig 1D). The percentage of SCLC patients with PSMA-positive NECs did not significantly differ from that of NSCLC patients and did not show significant correlation with age at diagnosis, gender, or clinical stage (limited and extensive stages) (data not shown). In addition, no detectable PSMA-positive lung cells were observed in normal lung tissues (0/33). Expression of both CD31 and PSMA was observed in the vascular endothelial cells of tumor tissues, whereas CD31 but not PSMA expression was observed in the vascular endothelial cells in normal lung tissues (Fig 1E).

## DISCUSSION

The main purpose of this study was to understand PSMA expression in various lung cancer tissues and to thus provide a scientific basis for the improvement of lung cancer diagnosis, treatment, and prognosis in the future. The highly specific PSMA expression in NECs may be associated with the high degree of atypia of tumor neovasculature. Angiogenesis is initiated by the activation of endothelial cells [21]. Tumor neovasculature differs from normal blood vessels in morphology, presenting a disorganized pattern with high tortuosity and incomplete pericyte coverage [22]. This mainly results from abnormal protein expression on the endothelial surface. The transcription of PSMA can be selectively activated through a transcriptional enhancer region in endothelial cells of the tumor neovasculature, but this region is absent in normal blood vessels [23]. Therefore, the selective expression of PSMA in tumor NECs may be activated by single or multiple tumor-secreted angiogenic factors [11].

The results of this study again confirmed that the NECs of NSCLC tissue specifically express PSMA (85.06%). Moreover, for the first time, we discovered that in the majority of SCLC patients (70.00%), NECs express PSMA. PSMA was specifically expressed in the NECs of lung cancers but not in normal blood vessels, suggesting that PSMA may serve as a new target for cancer therapy. In addition, this study found that PSMA was not only expressed in NECs of NSCLC but also in the tumor cells (in 54.02% of patients). To our knowledge, no related reports have yet been published. Our findings suggest that for NSCLC, a PSMA-targeted treatment strategy may have high therapeutic efficacy by simultaneously targeting both tumor cells and NECs.

The clinical significance of PSMA expression in tumor NECs is not fully understood. Currently, it is generally accepted that angiogenesis is critical for tumor growth, invasion, and metastasis [24]. However, our immunohistochemical results showed that the percentage of patients with PSMA-positive cells has no significant correlation with the size of the primary tumor mass or lymph node metastasis in NSCLC or SCLC, and the underlying reason for this needs to be further studied in the future. A significantly higher percentage of early-stage NSCLC patients had PSMA-positive endothelial cells compared with those with advanced NSCLC. This result is likely because a greater amount of neovasculature is present in early-stage tumors compared with late-stage tumors, where more tumor blood vessels have already undergone necrosis or maturation. It has been reported that PSMA expression in early-stage endometrial adenocarcinoma was significantly higher than that in advanced cancer, which is similar to the results of the present study [25]. Furthermore, we found that PSMA expression in NSCLC cancer cells was related to patient age, and the percentage of patients younger than 60 years with PSMA expression was significantly higher than that of patients 60 years of age or above. This result may be because younger patients have more robust cellular metabolism. The above two findings may have some clinical value for the diagnosis of NSCLC, and the detection of PSMA in NSCLC tissues may contribute to early diagnosis.

This study is the first report of PSMA expression in SCLC tumor NECs (in 70% of patients examined), and to our knowledge, no related reports have been published. In a manner significantly different from NSCLC, SCLC tumor cells did not express PSMA, and there was no detectable correlation between the percentage of SCLC patients with PSMA-positive tumor NECs and the clinical parameters investigated in this study. SCLC has different biological characteristics from NSCLC, which may impact PSMA expression.

In this study, we investigated the distribution of PSMA in lung cancer tissues. However, the clinical significance and value of PMSA expression remain to be clarified and require further in-depth investigation and evaluation. For example, this study revealed that more than half of NSCLC patients expressed PSMA in tumor cells but did not investigate the underlying mechanisms and clinical significance of this result. Moreover, the causes and clinical significance of the observation that there was a significant difference in PSMA expression between SCLC and NSCLC tissues needs to be further explored in future studies.

## Supporting Information

S1 Certificate The certificate about this study by the Ethics Committee of the First Affiliate Hospital of Dalian Medical University (KY2014-08). (PDF)

S2 Certificate The certificate about waiving the need for the written informed consent of used Paraffin-embedded samples by the ethics committee of the Tianjin Medical University Cancer Institute & Hospital (2012BWZ006). (PDF)

S3 Certificate The certificate of English Editing by American Journal Experts (AJE). (PDF)
