# Supplementary material for: Expression of Prostate-Specific Membrane Antigen in Lung Cancer Cells and Tumor Neovasculature Endothelial Cells and Its Clinical Significance
Source: PLoS One. 2015 May 15;10(5):e0125924. doi: 10.1371/journal.pone.0125924 (PMC4433228; doi:10.1371/journal.pone.0125924)
Supplement: S1 Certificate — (PDF) [file pone.0125924.s001.pdf]

大连医科大学附属第一医院医学伦理委员会批件

大连医科大学附属第一医院 <u>**肿瘤科**</u>**崔晓楠教授**递交的科研审查资料《肺癌细胞和新生血管内皮细胞中的前列腺特异性膜抗原及其临床意义》(审批编号：KY2014-08) 经过医院医学伦理委员会审核，符合医学伦理要求，同意该项目进行相关临床研究。

大连医科大学附属第一医院

医学伦理委员会

2014 年 2 月 10 日
