# Supplementary material for: Expression of Prostate-Specific Membrane Antigen in Lung Cancer Cells and Tumor Neovasculature Endothelial Cells and Its Clinical Significance
Source: PLoS One. 2015 May 15;10(5):e0125924. doi: 10.1371/journal.pone.0125924 (PMC4433228; doi:10.1371/journal.pone.0125924)
Supplement: S2 Certificate — (PDF) [file pone.0125924.s002.pdf]

2012BWKZ006

天津市滨海新区大港医院伦理委员会

王海龙

经伦理委员会审核后，批准开展滨海新区卫生局课题“rAAV 负载抗原基因转染 DC 治疗肺癌的研究（课题编号：2012BWKZ006）”，该课题第一部分研究 PSMA 在肺癌中的表达情况需使用肺癌病理蜡块标本可免除患者书面知情同意书的要求。

天津市滨海新区大港医院伦理委员会

主任：

2012 年 12 月 14 日
